# Investigation of the effect of nanoclay on the properties of quince seed mucilage edible films

**DOI:** 10.1002/fsn3.177

**Published:** 2014-10-17

**Authors:** Azadeh Sadat Shekarabi, Abdul Rasoul Oromiehie, Ali Vaziri, Mahdi Ardjmand, Ali Akbar Safekordi

**Affiliations:** 1 Department of Chemical Engineering, Science and Research Branch, Islamic Azad UniversityTehran, Iran; 2 Khatam Polymer Co.Units 9, No. 2, Hamsayeghan St. Valiaser St., Tehran, Iran; 3 Chemical Engineering Department, Islamic Azad Universities Tehran South BranchTehran, Iran

**Keywords:** Edible film, glass transition, nano clay, physical properties, quince seed mucilage

## Abstract

Some physical properties like Gas barrier, thermal stability, and mechanical properties and brittleness of pure biopolymers film are inadequate for food packaging. The functional properties of quince seed mucilage-based films were enhanced by addition of nanoclay (NC) (Cloisite 30B). Edible films were cast from heated aqueous solutions of quince seed (10% w/w) and NC (0.5%, 1%, 1.5%, and 2% w/w of quince seed). The effect of NC was studied in terms of tensile properties, water vapor permeability (WVP), oxygen permeability, and glass transition temperature (*T*_g_) of the nano composite films. In films containing NC, ultimate tensile strength enhanced to 22 MPa, and elongation increased from 2.48% to 6.5%. The addition of NC also improved gas barrier properties of the films. In films containing 2% NC, WVP decreased from 6.69 × 10^−7^ g·m^−1^·h^−1^·Pa^−1^ to 1.10 × 10^−7^ g·m^−1^·h^−1^·Pa^−1^ and oxygen permeability declined to 13.68 mL·day·m−^2^. NC also influences glass transition temperature significantly. The study demonstrated that the properties of quince seed mucilage edible films can be significantly improved using NC as reinforcement.

## Introduction

There is increased attention toward using degradable, renewable films and coatings from polysaccharide, protein, and lipid biopolymers. Natural biopolymer-based packaging materials have great prospective for extending food quality and safety as an innovative packaging and processing technology (Kokoszka et al. 2010). Use of biodegradable natural biopolymers reduces the amount of chemical wastes (Avila-Sosa et al. 2010). Although all edible films are not suitable barriers against water vapor, they can be used as a carrier of antimicrobial substances or preservatives, which protect food quality. In addition, edible films can be used to prolong the shelf-life and improve the characteristics, of food (Jimenez et al. 2010). The main film-forming materials are polysaccharides, proteins and lipids (Falguera et al. 2011); also the blend of these biopolymers with other biopolymers, glycerol, polyethylene glycol (as a plasticizer), hydrophobic substances and/or antimicrobial compounds has been widely used to improve the physical, organoleptic and nutritional properties of edible films (Vasconez et al. 2009). Potential applications of edible films and coatings have been widely reviewed (Falguera et al. 2011). In recent years, the market of edible films and coating has enjoyed significant growth which is expected to continue in future, so the search for better formulations of these films with improved characteristics from diverse sources is inevitable (Luduena et al. 2007). Several composites have been developed by adding reinforcing fillers to polymers to improve their thermal, mechanical, and barrier properties (Luduena et al. 2007). Quince (Cydonia oblonga Miller, Rosaceae family) is an important fruit species with high nutrient value and a positive influence on human health in Iran. The plant has been used in Iranian folk medicine for the treatment of a variety of diseases (Jouki et al. 2014). Nanoclay (NC), with high aspect ratio (100–1500) and extremely high surface-to-volume ratios (700–800 m^2^·g^−1^) present themselves as an excellent composite for improvement of the mechanical and barrier properties of polymers (Sinha Ray and Bousmina 2005). A uniform dispersion of nanoparticles leads to a very large filler interfacial area, which changes the molecular mobility and the physical properties of the material (Gontard et al. 1994; Dalmas et al. 2007). Exfoliated nanocomposites have been reported to exhibit the best properties due to the maximum clay–polymer interactions (Gontard et al. 1994). Clays is also reported to improve the mechanical strength of biopolymers (Alexandre et al. 2009). Incorporation of NC in polymer formulations enhances the tortuosity of the diffusive path for a penetrating molecule, and changes the molecular mobility, which enhances the thermal and mechanical properties amongst other advantages (Cyras et al. 2008; Marcelo Slavutsky et al. 2012). The cost was not evaluated at this stage, but is estimated to be quite low on a kg of fruit basis and well worth the protection it provides. Future work is planned which will evaluate the thickness of coating required for best protection, and when that data are available a more accurate cost analysis can be provided. In view of the above, the aim of this work was to evaluate the effect of different concentrations of NC added as a nano-reinforcing component on tensile properties, water vapor, oxygen permeability (O_2_P), and glass transition temperature of mucilage of quince seed (QSM) novel edible films.

## Material and Methods

### Material

Quince seeds were purchased from a local market (Tehran Province, Iran). Poly ethylene glycol 400 which was used as a plasticizer and Ethanol 96% were purchased from Merck Corporation. NC (Cloisite Na^+^) was obtained from Southern Clay (Gonzales, TX). Hand-held micrometer (Alton M820-25) with sensitivity of 0.01 mm was purchased from China. Glass vial was purchased from Caspian Company (Tehran, Iran).

### Method

#### Method of coating solutions preparation

About 10 g quince seeds were sieved (NO: 20) and washed with ethanol (96% w/v) for 5 min under constant stirring. Then ethanol was evaporated and seeds dried in an oven at 45°C. Aqueous QSM was extracted from whole seeds using distilled water (water to seed weight ratio of 25:1). The swelled seeds were then stirred at 1100 rpm, at 45°C for 15 min to scrape the mucilage layer off the seed surface. Next, the solutions were filtered. Film solution was prepared by slowly dissolving 10% mucilage, different levels of polyethylene glycol as a plasticizer (5% [w/w]) based on QSM weight was prepared under constant stirring (750 rpm) at 45°C for 15 min. Different concentrations of NC (ranging from 0.5 to 2 g per 100 g of QSM on a dry basis) were added to the solution. Finally, the emulsion was placed into an ultrasonic to remove air bubbles.

#### Method of film preparation

About 70 mL of the emulsion was poured on to teflon coated plates (40 × 40 cm) which was obtained from a local workshop in Iran. To control film thickness, the amount of solution poured was the same (300 mL) in each test, resulting in films with 0.08 ± 0.01 mm thickness, measured by a micrometer. We used a thin solution to avoid flow and viscous limitations, and used a carefully leveled table for each sample. Five determinations were made at random positions. The samples were then dried at 35°C in an oven to cast the films.

### Determination of physical properties of the films

#### Solubility in water

For this study, solubility in water was determined as the weight of the film that is dissolved after immersion in distilled water. A circular film sample was cut from each film, dried at 100 ± 2°C for 24 h in a laboratory oven, and weighed to determine the initial dry weight. The solubility in water of the different composite films was measured by immersion test in 50 mL of distilled water and stirred for 5 h at 25°C. After that period, the remaining pieces of film were taken out and dried at 100 ± 2°C until constant weight (final dry weight). The percentage of the total soluble matter (TSM) of the films was calculated using equation (1). TSM tests for each type of film were carried out in three replicates and average reported (Gontard et al. 1994).

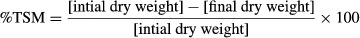
(1)

#### Water vapor permeability rate

Water vapor permeability (WVP) properties of the films were studied using the standard test method ASTM E 96 (ASTM E96-95 1995). Glass vials, with an average diameter of 0.8 mm and a depth of 2 cm, were accordingly used to determine WVP of films, instead of the standard cup***.*** The films were cut to a diameter slightly larger than the diameter of the vial into discs, and were covered by edible films with varying compositions. Each vial was placed in a desiccator containing saturated Mg (NO_3_)_2_·6H_2_O solution, which provided a constant RH of 52% and 25°C. The vials were weighed every 24 h and water vapor transport was determined by the weight loss of the vial. Changes in the weight of the vial were recorded as a function of time. Slopes were calculated by linear regression (weight change vs. time) and water vapor transmission rate (WVTR) was calculated by dividing the slope of the curve by the transfer area (m^2^) WVP (g·m^−1^·h^−1^·Pa^−1^) and calculated as (Saderi et al. 2005)

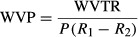
(2)
where *P* is the saturation vapor pressure of water (Pa) at the test temperature (25°C), *R*_1_ is the RH in the desiccator, *R*_2_, the RH in the vial and *X* is the film thickness (m). All measurements were performed in three replicates and the average reported.

#### Oxygen permeability O_2_P of films

O_2_P was determined based on the ASTM D3985-95 (ASTM D3985-3995 1995). O_2_P was measured at 25°C and 50 ± 1% RH according to the standard method D3985-95 (ASTM E96-95 1995). Each film was placed on a stainless steel mask with an open testing area of 0.0144 m^2^. Films were placed into the test cell and exposed to oxygen (O_2_) flow. Gases at similar absolute pressures and flow rates pass on either side of the sample film. An oxygen-rich gas, typically air or pure oxygen, passes on one side of the sample, while an oxygen-deficient carrier gas, such as nitrogen, passes on the opposite side. Oxygen permeates from the side with high concentration, through the film and into the oxygen-deficient carrier gas stream. After leaving the sample chamber, the carrier gas passes through an oxygen sensor to measure the oxygen concentration in the carrier gas. The analysis was performed in duplicate and the results were averaged (ASTM D3985-3995 1995).

#### Mechanical properties

Ultimate tensile strength (UTS) and strain to break (SB) of the films were determined at 24 ± 1°C and 52 ± 1% RH using a tensile tester (Elma, Tehran, Iran) according to the ASTM standard method D882-ASTM. Three dumbbell forms (10 × 1 cm) were cut from each of the samples and mounted between the machine grips. The initial grip separation and cross-head speed were set to 50 mm and 2 mm·min^−1^, respectively (ASTM D 882-891 1996).

#### Differential scanning calorimetry

The thermal properties of the films were carried out using a Differential Scanning Calorimeter (DSC) made by Setaram, France. The sample was placed into a sample pan of the DSC. Samples were scanned at a heating rate of 10°C/min between temperature ranges of −50°C and 150°C. Nitrogen was used as the purge gas at a flow rate of 20 mL·min^−1^. An empty aluminum pan was used as reference. In order to determine thermal properties, second heat ramps were used. All these properties were determined in duplicate and the results were averaged (Ryan et al. 2008).

#### Statistical analysis

The raw results of the tests were analyzed statistically by analysis of variance (ANOVA) procedure in SPSS (version 20, Chicago, IL) software. Duncan's multiple range test (*P* < 0.05) was used to detect differences among mean values of film properties.

## Result and Discussion

### Film solubility in water

Solubility in water is a major property of edible films that is related to the structural properties of film and the presence of components in the films, since potential applications may require water insolubility to enhance product integrity and water resistance. Table1 shows the effect of incorporating various concentrations of NC on the physical properties of QSM films. The amount of water present in composite films provides an indication of the films hydrophilicity, the more hydrophilic films being those that present the highest values of moisture content. At the same pH, films with greater NC content exhibited a lower solubility in water (*P* < 0.05). As can be seen in Table1, the incorporation of NC decreased water solubility of QSM films with respect to the control film. Addition of NC at a level of 2% w/w reduced the water solubility value. Marcelo et al. illustrated that water solubility of the film prepared by 5% clay and starch showed a significant decrease because of the interaction between the starch chains clay in nanocomposite films (Tunc and Osman 2007). Tunc and Osman obtained similar results for methylcellulose films (Ojagh et al. 2010). Furthermore, our results showed that the water solubility of QSM films reinforced with various concentrations of NC decreased significantly (*P* < 0.05) from 36.69% to 19.64% (Table1).

**Table 1:** Film solubility and moisture content of QSM films obtained with different NC concentrations

NC concentration (%w/w)	Thickness (mm)	Moisture content (%)	Solubility in water (%)
0	0.062	29.83	36.69
0.5	0.063	23.33	31.41
1	0.065	21.08	27.51
1.5	0.069	19.32	22.48
2	0.062	16.28	19.64

QSM, mucilage of quince seed; NC, nanoclay.

### Water vapor permeability

**Figure 1:**
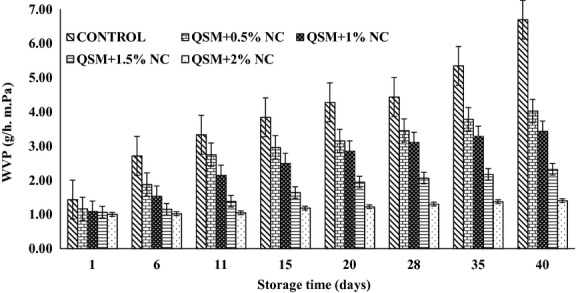
Effect of NC concentration on WVP of QSM films (*P* < 0.05). NC, nanoclay; WVP, water vapor permeability; QSM, mucilage of quince seed.

The WVP is the most important and extensive property of edible films because of its connections to degenerative reactions (Ojagh et al. 2010). Figure1 shows the WVP values of different composite films. The WVP of nano composite films changed significantly (*P* < 0.05) depending on the NC concentration used. When NC concentration increased from 0% to 2%, the WVP values decreased from 6.86 × 10^−7^ g·m^−1^·h^−1^·Pa^−1^ to 1.10 × 10^−7^ g·m^−1^·h^−1^·Pa^−1^ during a month. In fact, WVP of composite films declined significantly with the increase of NC, according to Figure1. WVP of the control films was 4.26 × 10^−7^ g·m^−1^·h^−1^·Pa^−1^ and decreased to 1.10 × 10^−7^ g·m^−1^·h^−1^·Pa^−1^ for 2% NC containing films. The hydrophobic or hydrophilic nature of biopolymers and presence of voids in their structure have a considerable influence on the WVP of resulting films (Ojagh et al. 2010). The film containing 2% NC exhibited the lowest WVP value. Decreased WVP by incorporation of NC was in agreement with the results reported for polymer blends which are studied for packaging applications (Ojagh et al. 2010). Tunc and coworkers obtained similar results for films made of wheat gluten and NC (Ojagh et al. 2010).

### Oxygen permeability O_2_P of the films

O_2_P of the QSM films with and without NC is summarized in Table2. The O_2_P of the control QSM film was 30.81 ± 0.138 mL·day·m^−2^, by increasing NC to 2% w/w this value decreased to 13.68 ± 0.263 mL·day·m^−2^, resulting in better oxygen barrier properties of QSM films. The films without NC exhibited the highest values of O_2_P among all films tested (*P* < 0.05). These results, as with WVP values, could be due to the hydrophilic nature of NC. The same result was previously observed in the behavior of master batch-based nano composite, that is, higher levels of NC results in lower O_2_P (Acosta et al. 2013).

**Table 2:** Effect of NC concentration on oxygen permeability of QSM films (*P* < 0.05)

NC concentration (%w/w)	Thickness (mm)	Oxygen permeability (mL·day·m^−2^)
0	0.062	30.81
0.5	0.063	23.27
1	0.065	19.64
1.5	0.064	14.58
2	0.062	13.68

QSM, mucilage of quince seed; NC, nanoclay.

### Tensile strength and percentage of elongation

**Figure 2:**
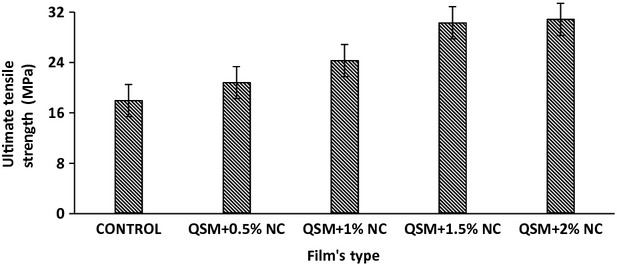
Ultimate tensile strength (UTS) of the QSM films as a function of NC (*P* < 0.05). NC, nanoclay; QSM, mucilage of quince seed.

**Figure 3:**
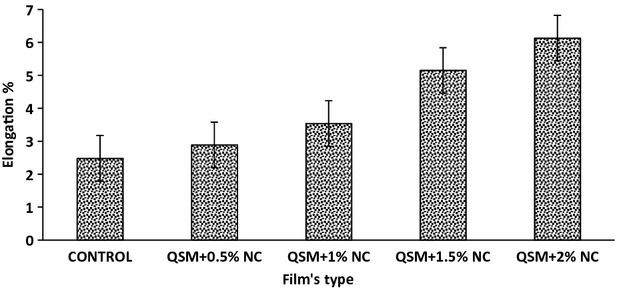
Elongation at break of the QSM films as a function of NC (*P* < 0.05). QSM, mucilage of quince seed; NC, nanoclay.

Tensile strength and percentage of elongation are two significant properties in packaging material. Effect of NC concentration on tensile properties of pure QSM and QSM/clay nanocomposite films was investigated. Figures2, 3 show the relationships between NC content and the tensile properties of the QSM films. Improvement was seen by addition of NC. The results show improvement of mechanical strength with the increase of NC. According to these figures, NC addition of up to 2% increases the mechanical parameters (UTS and %E) for all films compared to the control. In control film, the UTS and elongation were 21.56 MPa and 6.34% and in the composite film with 2% NC there were 31.84 MPa and 2.37%, respectively. The films which did not contain NC had a poor result, In general, the composite film with (2% w/w) NC had higher (*P* < 0.05) values of this parameter. It seems that the addition of NC improves film strength due to the strong interaction between NC and QSM, resulting from a reduced-free volume and molecular mobility of the polymer (Ghasemlou et al. 2011). This is in agreement with the results reported by Pasbakhsh and coworkers who found that by incorporation of 5 (w/w%) of HNTs, Young's modulus and tensile strength improved by 21% and 34%, respectively (De Silva et al. 2013). They found that the addition of Halloysite NC as an environmentally friendly nanofiller to chitosan biopolymer can increase the applications of this biopolymer, especially when the mechanical properties are concerned (De Silva et al. 2013).

### Thermal properties

The effect of NC concentration in QSM films on the thermal properties was studied by DSC. The initial temperature of degradation, temperature at the maximum degradation rate and apparent enthalpy were measured using the first DSC scan for all the films. Below *T*_g_, films are rigid and brittle, whereas above it films become flexible (Altoik et al. 2010). Table3 shows the glass transition temperatures (*T*_g_) and melting temperatures (*T*_m_) of the QSM-based films with different NC concentrations. The glass transition temperature (*T*_g_) is the temperature at which the material undergoes a structural transition from a glassy state to a more viscous rubbery state (Ojagh et al. 2010). The findings of this study indicate that control films (QSM) had a *T*_g_ value of about (13.5°C), *T*_m_ (76.38°C) and Δ*H* of (92.23). Incorporating NC (0% to 2% w/w) into the QSM films significantly increased *T*_g_, *T*_m,_ and Δ*H*. Nano clay in QSM films makes films more hydrophilic and maintains a higher moisture content compared to control films when conditioned at the same humidity (RH %) and temperature. Composite film containing 2% NC had a *T*_g_ value of about (83 ± 0.7°C), *T*_m_ (130 ± 0.5°C) and Δ*H* of (115.48). Significant increase in *T*_g_ values with increasing NC content, shows that the NC particles create strong bonding between the polymer chains. This limits the mobility of polymer chains and therefore requires higher temperatures for the movement of chains.

**Table 3:** Differential scanning calorimetry (DSC) measurement results of QSM films with different NC concentrations

NC concentration (%w/w)	*T*_g_ (°C)	Δ*H*_f_ (J·g^−1^)
0.0	13.5	92.23
0.5	28.47	97.48
1.0	63.82	103.35
1.5	76.38	109.27
2.0	83.4	115.48

QSM, mucilage of quince seed; NC, nanoclay.

## Conclusion

The properties of QSM-based films were enhanced by the addition of NC. These research results showed that the QSM films incorporated with 2% NC have a great potential for application as a natural film to preserve food.

Water solubility, gas permeability, mechanical and thermal properties of QSM-based nanocomposite films varied depending on the NC concentrations. Addition of NC at a level of 2% w/w reduced the water solubility from 36.69% to 19.64%. Hydrophilic natural montmorillonite, Cloisite Na^+^ showed suitable interaction with the QSM matrix. Films incorporated with 2% NC showed in significant increased tensile strength and elongation of the films. Moreover, the film prepared with the 2% w/w NC was found to be the best as it had the lowest water vapor and O_2_P. WVP and O_2_P of the composite film containing 2% NC decreased to 1.10 × 10^−7^ g·m^−1^·h^−1^·Pa^−1^ and 13.68 mL·day·m^−2^. Moreover, the tensile strength and elongation at break rose to 22 MPa and 6.5%, respectively, in films with 2% NC. Furthermore, glass transition temperatures of QSM film containing 2% NC increased to 83.4°C compared to control films. These results suggest that the addition of NC as an environmentally friendly nanofiller to QSM biopolymer can increase the applications and physical properties of this biopolymer.
